# Isopropyl­triphenyl­phospho­nium bromide monohydrate

**DOI:** 10.1107/S1600536811044849

**Published:** 2011-11-02

**Authors:** Hai Wang, Xi-Man Zhang, Ping Li, Hong-Yu Chen

**Affiliations:** a School of Chemistry and Chemical Engineering, TaiShan Medical University, Tai’an 271016, People’s Republic of China

## Abstract

In the title water-solvated salt, C_21_H_22_P^+^·Br^−^·H_2_O, the ionic components are linked by short C—H⋯Br contacts along the *a*-axis direction. The two half occupied water mol­ecules are connected to each other by strong O—H⋯O hydrogen bonds and they are also linked to the bromide anion by short O—H⋯Br contacts.

## Related literature

For information on phase-transfer catalysts, see: Asai *et al.* (1994 ▶). For the crystal structure of tetra­phenyl­phospho­niuum bromide, see: Alcock *et al.* (1985 ▶). For standard bond lengths, see: Allen *et al.* (1987 ▶).
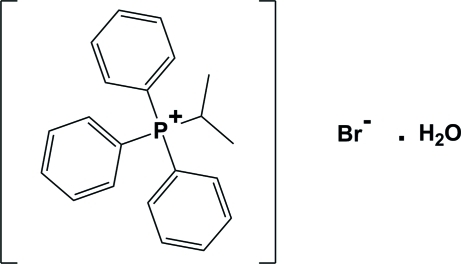


## Experimental

### 

#### Crystal data

C_21_H_22_P^+^·Br^−^·H_2_O
*M* *_r_* = 403.28
Orthorhombic, 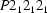

*a* = 9.078 (5) Å
*b* = 13.043 (5) Å
*c* = 17.755 (5) Å
*V* = 2102.3 (15) Å^3^
*Z* = 4
Mo *K*α radiation
μ = 2.04 mm^−1^
*T* = 293 K
0.20 × 0.15 × 0.13 mm

#### Data collection

Brucker APEXII CCD area-detector diffractometer
Absorption correction: multi-scan (*SADABS*; Sheldrick, 1996 ▶) *T*_min_ = 0.668, *T*_max_ = 0.688
12177 measured reflections
4250 independent reflections
3331 reflections with *I* > 2σ(*I*)
*R*_int_ = 0.034

#### Refinement

*R*[*F* ^2^ > 2σ(*F* ^2^)] = 0.049
*wR*(*F* ^2^) = 0.140
*S* = 1.03
4250 reflections
228 parameters
6 restraints
H-atom parameters constrained
Δρ_max_ = 0.82 e Å^−3^
Δρ_min_ = −0.28 e Å^−3^
Absolute structure: Flack (1983 ▶), 1799 Friedel pairs
Flack parameter: 0.014 (13)

### 

Data collection: *APEX2* (Bruker, 2005 ▶); cell refinement: *SAINT* (Bruker, 2005 ▶); data reduction: *SAINT*; program(s) used to solve structure: *SHELXS97* (Sheldrick, 2008 ▶); program(s) used to refine structure: *SHELXL97* (Sheldrick, 2008 ▶); molecular graphics: *SHELXTL* (Sheldrick, 2008 ▶); software used to prepare material for publication: *SHELXTL*.

## Supplementary Material

Crystal structure: contains datablock(s) I, global. DOI: 10.1107/S1600536811044849/su2327sup1.cif

Structure factors: contains datablock(s) I. DOI: 10.1107/S1600536811044849/su2327Isup2.hkl

Supplementary material file. DOI: 10.1107/S1600536811044849/su2327Isup3.cml

Additional supplementary materials:  crystallographic information; 3D view; checkCIF report

## Figures and Tables

**Table 1:** Hydrogen-bond geometry (Å, °)

*D*—H⋯*A*	*D*—H	H⋯*A*	*D*⋯*A*	*D*—H⋯*A*
O1—H1*O*1⋯Br1^i^	0.85	2.86	3.701 (8)	169
O1—H2*O*1⋯O2	0.85	2.20	2.960 (11)	149
O2—H1*O*2⋯Br1^ii^	0.85	2.68	3.526 (7)	175
O2—H2*O*2⋯Br1^iii^	0.85	2.81	3.598 (8)	154
C19—H19⋯Br1^iv^	0.98	2.76	3.723 (4)	169

Symmetry codes: (i) 
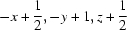
; (ii) 
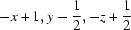
; (iii) 
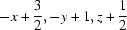
; (iv) 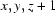.

**Fig. 1.**
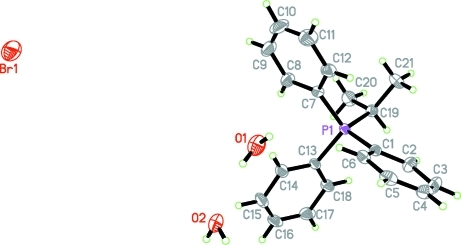
A view of the molecular structure of the title compound, with the numbering scheme and thermal ellipsoids drawn at the 30%probability level.

**Fig. 2.**
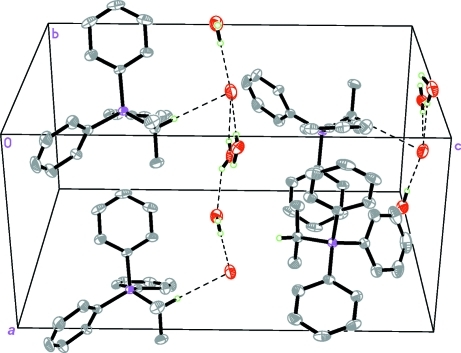
A view of the crystal packing of the title compound, showing the O-H···Br and O-H···O hydrogen bonds, and the C-H···Br contacts as dashed lines [H atoms not involved in these contacts have been omitted for calrity].

## supplementary crystallographic information

### Comment

The title compound belongs to a family of phase transfer catalysts, which usually contain large alkyl or aryl ions, such as *R*_4_N^+^,*R*_4_P^+^,*R*_4_B^-^*etc*. Because of their ease of dissolution in water, such salts have a conveninient to satisfactory hydrophobic hydration(Asai, *et al.*, 1994).

The asymmetry unit of the title structure consists of one isopropyltriphenylphosphonium cation, one bromide anion and two half occupied water molecules (Fig. 1). In contrast with tetraphenylphosphonium salts, the major character of the cation of the title compound is that one phenyl group has been substituted by an isopropyl group. The fluctuation of the C_phenyl_—P1 bond lengths in the title compound [from 1.789 (4)–1.806 (4) Å] is similar to that in the crystal structure of tetraphenylphosphonium bromide [1.801 (3)Å; Alcock, *et al.*, 1985]. The bonds between P1 and the C atoms of phenyl rings are C*sp*^2^—Psp^3^ bonds, but the connection between P1 and isopropyl group is typically an C*sp*^3^—Psp^3^ bond (Allen, *et al.*, 1987). The bond length of C19—P1 [1.818 (3) Å] associated with the isopropyl group is longer than that involving the phenyl groups, which vary from 1.789 (4) - 1.806 (4) Å.

In the crystal the water molecules are linked to the Br anion by short O—H···Br contacts, and the two half occupied water molecules are connected to one another by strong O-H···O hydrogen bonds (Table 1 and Fig. 2). The large cations and bromide anions are linked by short C19—H19···Br1 contacts (Table 1 and Fig. 2). These weak interactions also that play an important role in the stabilization of the crystal structure.

### Experimental

Triphenyl phosphine (10.5 g) and 2-bromopropane (4.2 mL) were placed in a teflon lined tube. The sealed tube was placed in an autoclave and heated to 433 K for 48 h, then cooled at a rate of 10 K/min. Colourless block-like crystals of the title compound were obtained.

### Refinement

The water H atoms were located in difference Fourier maps and were subsequently treated as riding atoms: O—H = 0.85 Å with U_iso_(H) = 1.5U_eq_(O). The C-bound H-atoms were included in calculated positions and treated as riding atoms: C-H = 0.93, 0.96, and 0.98 Å for CH(aromatic), CH_3_, and CH(methine) H-atoms, respectively, with = k × U_eq_(parent C-atom), where k = 1.5 for CH_3_ H-atoms and k = 1.2 for all other H-atoms.

### Crystal data

**Table:**

C_21_H_22_P^+^·Br^−^·H_2_O	*F*(000) = 832
*M**_r_* = 403.28	*D*_x_ = 1.274 Mg m^−^^3^
Orthorhombic, *P*2_1_2_1_2_1_	Mo *K*α radiation, λ = 0.71069 Å
Hall symbol: P 2ac 2ab	Cell parameters from 3565 reflections
*a* = 9.078 (5) Å	θ = 2.5–23.5°
*b* = 13.043 (5) Å	µ = 2.04 mm^−^^1^
*c* = 17.755 (5) Å	*T* = 293 K
*V* = 2102.3 (15) Å^3^	ROD, colourless
*Z* = 4	0.20 × 0.15 × 0.13 mm

### Data collection

**Table:**

Brucker APEXII CCD area-detector diffractometer	4250 independent reflections
Radiation source: fine-focus sealed tube	3331 reflections with *I* > 2σ(*I*)
graphite	*R*_int_ = 0.034
phi and ω scans	θ_max_ = 26.4°, θ_min_ = 1.9°
Absorption correction: multi-scan (*SADABS*; Sheldrick, 1996)	*h* = −7→11
*T*_min_ = 0.668, *T*_max_ = 0.688	*k* = −16→15
12177 measured reflections	*l* = −21→21

### Refinement

**Table:**

Refinement on *F*^2^	Secondary atom site location: difference Fourier map
Least-squares matrix: full	Hydrogen site location: inferred from neighbouring sites
*R*[*F*^2^ > 2σ(*F*^2^)] = 0.049	H-atom parameters constrained
*wR*(*F*^2^) = 0.140	*w* = 1/[σ^2^(*F*_o_^2^) + (0.0853*P*)^2^] where *P* = (*F*_o_^2^ + 2*F*_c_^2^)/3
*S* = 1.03	(Δ/σ)_max_ = 0.001
4250 reflections	Δρ_max_ = 0.82 e Å^−^^3^
228 parameters	Δρ_min_ = −0.28 e Å^−^^3^
6 restraints	Absolute structure: Flack (1983), 1799 Friedel pairs
Primary atom site location: structure-invariant direct methods	Flack parameter: 0.014 (13)

### Special details

**Table:**

Geometry. All e.s.d.'s (except the e.s.d. in the dihedral angle between two l.s. planes) are estimated using the full covariance matrix. The cell e.s.d.'s are taken into account individually in the estimation of e.s.d.'s in distances, angles and torsion angles; correlations between e.s.d.'s in cell parameters are only used when they are defined by crystal symmetry. An approximate (isotropic) treatment of cell e.s.d.'s is used for estimating e.s.d.'s involving l.s. planes.
Refinement. Refinement of *F*^2^ against ALL reflections. The weighted *R*-factor *wR* and goodness of fit *S* are based on *F*^2^, conventional *R*-factors *R* are based on *F*, with *F* set to zero for negative *F*^2^. The threshold expression of *F*^2^ > σ(*F*^2^) is used only for calculating *R*-factors(gt) *etc*. and is not relevant to the choice of reflections for refinement. *R*-factors based on *F*^2^ are statistically about twice as large as those based on *F*, and *R*- factors based on ALL data will be even larger.

### Fractional atomic coordinates and isotropic or equivalent isotropic displacement parameters (Å^2^)

**Table:**

	*x*	*y*	*z*	*U*_iso_*/*U*_eq_	Occ. (<1)
Br1	0.46181 (8)	0.58731 (5)	0.00805 (3)	0.0720 (2)	
C1	0.2804 (4)	0.5002 (3)	0.7564 (2)	0.0320 (8)	
C2	0.2503 (5)	0.4645 (3)	0.8289 (3)	0.0468 (11)	
H2	0.2722	0.5050	0.8705	0.056*	
C3	0.1873 (6)	0.3679 (4)	0.8386 (3)	0.0570 (13)	
H3	0.1690	0.3433	0.8868	0.068*	
C4	0.1527 (5)	0.3098 (3)	0.7778 (3)	0.0502 (13)	
H4	0.1075	0.2466	0.7850	0.060*	
C5	0.1833 (5)	0.3426 (3)	0.7054 (3)	0.0463 (12)	
H5	0.1607	0.3011	0.6644	0.056*	
C6	0.2478 (4)	0.4378 (3)	0.6943 (2)	0.0371 (9)	
H6	0.2694	0.4602	0.6458	0.045*	
C7	0.3207 (5)	0.6876 (3)	0.6626 (2)	0.0340 (9)	
C8	0.4105 (6)	0.7668 (3)	0.6355 (3)	0.0493 (12)	
H8	0.5032	0.7770	0.6564	0.059*	
C9	0.3631 (8)	0.8296 (4)	0.5783 (3)	0.0737 (18)	
H9	0.4228	0.8822	0.5604	0.088*	
C10	0.2238 (10)	0.8130 (5)	0.5476 (3)	0.082 (2)	
H10	0.1893	0.8561	0.5098	0.098*	
C11	0.1386 (7)	0.7354 (6)	0.5719 (3)	0.0754 (18)	
H11	0.0478	0.7239	0.5491	0.090*	
C12	0.1835 (5)	0.6726 (4)	0.6299 (3)	0.0498 (12)	
H12	0.1223	0.6205	0.6471	0.060*	
C13	0.5706 (4)	0.5878 (3)	0.7380 (2)	0.0312 (8)	
C14	0.6312 (5)	0.5673 (3)	0.6682 (3)	0.0430 (10)	
H14	0.5765	0.5766	0.6245	0.052*	
C15	0.7767 (5)	0.5321 (3)	0.6648 (3)	0.0538 (14)	
H15	0.8199	0.5187	0.6183	0.065*	
C16	0.8552 (5)	0.5173 (3)	0.7293 (4)	0.0567 (14)	
H16	0.9514	0.4930	0.7264	0.068*	
C17	0.7943 (5)	0.5379 (4)	0.7989 (3)	0.0569 (14)	
H17	0.8493	0.5277	0.8425	0.068*	
C18	0.6508 (4)	0.5737 (3)	0.8037 (3)	0.0446 (11)	
H18	0.6089	0.5880	0.8503	0.054*	
C19	0.3403 (5)	0.7009 (3)	0.8260 (2)	0.0352 (9)	
H19	0.3655	0.6618	0.8713	0.042*	
C20	0.4348 (6)	0.7974 (3)	0.8257 (3)	0.0538 (13)	
H20A	0.4054	0.8408	0.7847	0.081*	
H20B	0.5365	0.7789	0.8198	0.081*	
H20C	0.4220	0.8334	0.8724	0.081*	
C21	0.1779 (5)	0.7266 (4)	0.8303 (3)	0.0530 (12)	
H21A	0.1593	0.7669	0.8745	0.079*	
H21B	0.1216	0.6644	0.8327	0.079*	
H21C	0.1498	0.7648	0.7864	0.079*	
O1	0.4288 (9)	0.4940 (6)	0.5103 (4)	0.0650 (19)	0.50
H1O1	0.3405	0.4745	0.5027	0.097*	0.50
H2O1	0.4722	0.4469	0.4855	0.097*	0.50
O2	0.6608 (8)	0.3426 (5)	0.4736 (4)	0.0566 (18)	0.50
H1O2	0.6370	0.2797	0.4777	0.085*	0.50
H2O2	0.7522	0.3365	0.4835	0.085*	0.50
P1	0.37744 (10)	0.61951 (7)	0.74506 (6)	0.0269 (2)	

### Atomic displacement parameters (Å^2^)

**Table:**

	*U*^11^	*U*^22^	*U*^33^	*U*^12^	*U*^13^	*U*^23^
Br1	0.0904 (5)	0.0823 (4)	0.0433 (3)	0.0170 (3)	−0.0098 (3)	0.0009 (3)
C1	0.0245 (18)	0.0290 (17)	0.042 (2)	0.0013 (15)	0.0014 (18)	0.0014 (17)
C2	0.054 (3)	0.039 (2)	0.047 (3)	−0.008 (2)	0.000 (2)	0.005 (2)
C3	0.062 (3)	0.048 (3)	0.061 (3)	−0.016 (3)	0.000 (3)	0.019 (2)
C4	0.035 (3)	0.032 (2)	0.084 (4)	−0.0057 (18)	−0.004 (2)	0.004 (2)
C5	0.035 (2)	0.035 (2)	0.068 (3)	−0.001 (2)	−0.006 (2)	−0.018 (2)
C6	0.033 (2)	0.037 (2)	0.041 (2)	0.0025 (18)	0.0034 (18)	−0.0061 (18)
C7	0.033 (2)	0.032 (2)	0.036 (2)	0.0060 (17)	0.0020 (18)	0.0009 (17)
C8	0.059 (3)	0.040 (2)	0.049 (3)	0.000 (2)	0.006 (2)	0.006 (2)
C9	0.111 (5)	0.050 (3)	0.061 (4)	0.020 (3)	0.026 (4)	0.021 (3)
C10	0.125 (6)	0.077 (4)	0.042 (3)	0.043 (4)	−0.001 (4)	0.021 (3)
C11	0.071 (4)	0.110 (5)	0.045 (3)	0.026 (4)	−0.017 (3)	0.006 (3)
C12	0.043 (3)	0.064 (3)	0.043 (3)	0.014 (2)	−0.006 (2)	0.005 (2)
C13	0.0247 (17)	0.0274 (17)	0.042 (2)	0.0007 (14)	0.0065 (16)	−0.0011 (17)
C14	0.041 (2)	0.036 (2)	0.053 (3)	0.000 (2)	0.009 (2)	−0.0011 (19)
C15	0.041 (3)	0.042 (3)	0.079 (4)	0.000 (2)	0.028 (3)	−0.007 (3)
C16	0.029 (2)	0.038 (2)	0.104 (5)	0.0034 (19)	0.016 (3)	0.006 (3)
C17	0.035 (3)	0.053 (3)	0.083 (4)	0.003 (2)	−0.007 (3)	0.016 (3)
C18	0.030 (2)	0.049 (3)	0.055 (3)	0.006 (2)	0.0002 (19)	0.004 (2)
C19	0.037 (2)	0.032 (2)	0.036 (2)	0.0005 (17)	0.0023 (18)	−0.0023 (16)
C20	0.053 (3)	0.037 (2)	0.071 (3)	−0.009 (2)	0.008 (3)	−0.010 (2)
C21	0.036 (2)	0.061 (3)	0.061 (3)	0.009 (2)	0.006 (2)	−0.012 (2)
O1	0.072 (5)	0.077 (5)	0.045 (4)	−0.015 (4)	−0.011 (4)	0.001 (3)
O2	0.062 (4)	0.059 (4)	0.049 (4)	−0.009 (3)	0.008 (3)	0.013 (3)
P1	0.0222 (4)	0.0273 (4)	0.0313 (5)	0.0001 (4)	0.0016 (4)	0.0009 (4)

### Geometric parameters (Å, °)

**Table:**

C1—C2	1.395 (6)	C13—C18	1.387 (6)
C1—C6	1.402 (6)	C13—P1	1.806 (4)
C1—P1	1.800 (4)	C14—C15	1.400 (6)
C2—C3	1.394 (6)	C14—H14	0.9300
C2—H2	0.9300	C15—C16	1.363 (8)
C3—C4	1.355 (7)	C15—H15	0.9300
C3—H3	0.9300	C16—C17	1.381 (8)
C4—C5	1.384 (7)	C16—H16	0.9300
C4—H4	0.9300	C17—C18	1.386 (6)
C5—C6	1.387 (6)	C17—H17	0.9300
C5—H5	0.9300	C18—H18	0.9300
C6—H6	0.9300	C19—C21	1.514 (6)
C7—C12	1.388 (6)	C19—C20	1.523 (6)
C7—C8	1.400 (6)	C19—P1	1.818 (4)
C7—P1	1.789 (4)	C19—H19	0.9800
C8—C9	1.374 (7)	C20—H20A	0.9600
C8—H8	0.9300	C20—H20B	0.9600
C9—C10	1.394 (10)	C20—H20C	0.9600
C9—H9	0.9300	C21—H21A	0.9600
C10—C11	1.345 (10)	C21—H21B	0.9600
C10—H10	0.9300	C21—H21C	0.9600
C11—C12	1.377 (7)	O1—H1O1	0.8522
C11—H11	0.9300	O1—H2O1	0.8527
C12—H12	0.9300	O2—H1O2	0.8515
C13—C14	1.382 (6)	O2—H2O2	0.8512
			
C2—C1—C6	119.4 (4)	C13—C14—H14	120.7
C2—C1—P1	119.2 (3)	C15—C14—H14	120.7
C6—C1—P1	121.1 (3)	C16—C15—C14	120.2 (5)
C3—C2—C1	119.7 (5)	C16—C15—H15	119.9
C3—C2—H2	120.2	C14—C15—H15	119.9
C1—C2—H2	120.2	C15—C16—C17	121.1 (4)
C4—C3—C2	120.2 (5)	C15—C16—H16	119.5
C4—C3—H3	119.9	C17—C16—H16	119.5
C2—C3—H3	119.9	C16—C17—C18	119.7 (5)
C3—C4—C5	121.4 (4)	C16—C17—H17	120.1
C3—C4—H4	119.3	C18—C17—H17	120.1
C5—C4—H4	119.3	C17—C18—C13	119.2 (5)
C4—C5—C6	119.5 (4)	C17—C18—H18	120.4
C4—C5—H5	120.2	C13—C18—H18	120.4
C6—C5—H5	120.2	C21—C19—C20	111.4 (4)
C5—C6—C1	119.8 (4)	C21—C19—P1	110.5 (3)
C5—C6—H6	120.1	C20—C19—P1	112.1 (3)
C1—C6—H6	120.1	C21—C19—H19	107.6
C12—C7—C8	118.9 (4)	C20—C19—H19	107.6
C12—C7—P1	122.0 (3)	P1—C19—H19	107.6
C8—C7—P1	118.7 (3)	C19—C20—H20A	109.5
C9—C8—C7	120.8 (5)	C19—C20—H20B	109.5
C9—C8—H8	119.6	H20A—C20—H20B	109.5
C7—C8—H8	119.6	C19—C20—H20C	109.5
C8—C9—C10	118.7 (6)	H20A—C20—H20C	109.5
C8—C9—H9	120.7	H20B—C20—H20C	109.5
C10—C9—H9	120.7	C19—C21—H21A	109.5
C11—C10—C9	120.9 (5)	C19—C21—H21B	109.5
C11—C10—H10	119.6	H21A—C21—H21B	109.5
C9—C10—H10	119.6	C19—C21—H21C	109.5
C10—C11—C12	121.1 (6)	H21A—C21—H21C	109.5
C10—C11—H11	119.4	H21B—C21—H21C	109.5
C12—C11—H11	119.4	H1O1—O1—H2O1	98.0
C11—C12—C7	119.6 (5)	H1O2—O2—H2O2	98.0
C11—C12—H12	120.2	C7—P1—C1	112.38 (19)
C7—C12—H12	120.2	C7—P1—C13	109.67 (19)
C14—C13—C18	121.2 (4)	C1—P1—C13	106.56 (17)
C14—C13—P1	119.5 (3)	C7—P1—C19	107.68 (18)
C18—C13—P1	118.8 (3)	C1—P1—C19	108.99 (19)
C13—C14—C15	118.6 (5)	C13—P1—C19	111.62 (19)

### Hydrogen-bond geometry (Å, °)

**Table:**

*D*—H···*A*	*D*—H	H···*A*	*D*···*A*	*D*—H···*A*
O1—H1O1···Br1^i^	0.85	2.86	3.701 (8)	169.
O1—H2O1···O2	0.85	2.20	2.960 (11)	149.
O2—H1O2···Br1^ii^	0.85	2.68	3.526 (7)	175.
O2—H2O2···Br1^iii^	0.85	2.81	3.598 (8)	154.
C19—H19···Br1^iv^	0.98	2.76	3.723 (4)	169.

Symmetry codes: (i) −*x*+1/2, −*y*+1, *z*+1/2; (ii) −*x*+1, *y*−1/2, −*z*+1/2; (iii) −*x*+3/2, −*y*+1, *z*+1/2; (iv) *x*, *y*, *z*+1.
